# Psychosis in Alzheimer’s Disease

**DOI:** 10.1007/s11910-020-01074-y

**Published:** 2020-10-13

**Authors:** Clive Ballard, Helen C. Kales, Constantine Lyketsos, Dag Aarsland, Byron Creese, Roger Mills, Hilde Williams, Robert A. Sweet

**Affiliations:** 1 The University of Exeter Medical School, College of Medicine and Health, The University of Exeter, St Luke’s Campus, Magdalen Road, Exeter, EX1 2LU UK; 2 University of Michigan, Ann Arbor, MI USA; 3 Johns Hopkins University, Baltimore, MD USA; 4 University Hospital Stavanger, Stavanger, Norway; 5 King’s College London, London, UK; 6 University of Pittsburgh, Pittsburgh, PA USA

**Keywords:** Alzheimer’s disease, Psychosis, Frequency, Impact, Mechanisms, Non-pharmacological, Pharmacological

## Abstract

**Purpose of Review:**

To review the incidence, treatment and genetics of psychosis in people with mild cognitive impairment (MCI) and Alzheimer’s disease (AD).

**Recent Findings:**

Psychosis in Alzheimer’s disease (AD) has an incidence of ~ 10% per year. There is limited evidence regarding psychological interventions. Pharmacological management has focused on atypical antipsychotics, balancing modest benefits with evidence of long-term harms. The 5HT2A inverse agonist pimavanserin appears to confer benefit in PD psychosis with initial evidence of benefit in AD. Cholinesterase inhibitors give modest benefits in DLB psychosis. The utility of muscarinic agonists, lithium, glutamatergic and noradrenergic modulators needs further study.

**Summary:**

Recent work has confirmed the importance of psychosis in MCI as well as AD. The lack of evidence regarding psychological therapies is an urgent knowledge gap, but there is encouraging evidence for emerging pharmacological treatments. Genetics will provide an opportunity for precision medicine and new treatment targets.

## Introduction

Psychosis occurs in the majority of people with Alzheimer’s disease over the course of their illness. It is distressing for patients and caregivers and is associated with an accelerated trajectory of decline and dependency. Recent work has focussed on the presence of psychosis in people with mild cognitive impairment (MCI), as a risk factor for the development of Alzheimer’s disease. Over the last few years, studies have also begun to identify novel risk factors, and there have been promising RCTs of new drug therapies. A review is therefore extremely timely.

## Background

### Frequency and Course

There are 45 million people with dementia worldwide, the majority of whom have Alzheimer’s disease (AD) [1]. While AD is commonly thought of as a memory disorder, behavioural and psychological symptoms of dementia (BPSD) are nearly universal and include psychotic symptoms. Over half of people with AD will experience psychotic symptoms during their illness [2, 3]. Systematic reviews indicate a cross-sectional prevalence of about 40%, though lower rates in community populations and higher rates in clinical settings are recognized. Notably, most prevalence studies reflect populations with a mean MMSE indicating moderate AD severity [3–6]. Subsequent work has confirmed a lower frequency of psychosis in mild AD, with one US study of over 6000 people reporting a prevalence of 12% for delusions and 3% for hallucinations [7]. Subsequent studies evaluating psychosis in mild cognitive impairment (MCI) demonstrate a prevalence higher than age-matched controls and lower than people with AD [8]. The two largest studies suggest a 3–5% frequency of delusions and 1% hallucinations in MCI [7, 9]. Sub-syndromal delusions and hallucinations are also present in cognitively normal older adults, at a frequency of around 3% and less than 1% respectively, and may represent early marker cognitive decline [10, 11•].

In dementia, once present, psychotic symptoms commonly persist for at least a year in two-thirds of people [12]. A follow-up study reported a 2-year persistence of delusions and hallucinations in 43% and 73% of people, respectively [2]. However, this may conceal an underlying pattern of remission and recurrence. For example, in 125 people with AD followed monthly for 12 months, 30 (54%) had resolution of symptoms over 3 months without specific treatment, but among these, eight (27%) subsequently relapsed in the first year [13]. Over the last 15 years, studies have consistently highlighted the impact of psychotic symptoms in MCI, predicting greater cognitive decline and more rapid conversion to AD or other dementias [14–16].

## Definition and Diagnosis

Psychosis is defined by the presence of delusions, delusional misidentification and hallucinations. Although, superficially, this suggests similarity to functional psychotic disorders such as schizophrenia, the phenomenology of psychosis in dementia is very different. Delusions, present in around 35% of people, are usually simple; common symptoms include delusions of theft, persecution, infidelity, abandonment or that deceased relatives are still living [17, 18]. Other delusions in AD are related to misidentification such as beliefs that one’s home is not one’s home; that a family member is someone else, has been duplicated or is an imposter (Capgras delusion); or that someone is living in the house (phantom boarder delusion) [19]. Even more common symptoms include misperceiving television, mirror or photographic images as real people or objects.

Hallucinations are one of the most frequent psychotic symptoms in AD with a median prevalence of 23% across studies [14]. Hallucinations can occur in any sensory modality, but visual hallucinations are the most frequent, followed by auditory [4]. Typical hallucinatory symptoms of schizophrenia such as hearing multiple voices talking to one another or running a commentary on the person’s actions are extremely rare in AD.

The most widely used diagnostic criteria are those proposed by Jeste and Finkel [4]. In addition to the presence of psychotic symptoms, the criteria require clarification that the psychotic symptoms are not part of a delirium or functional psychosis and did not pre-date AD onset. Symptoms should also have been present for at least a month and result in functional disability on the individual or others.

Diagnostic criteria and many clinical trials consider psychosis as a unitary syndrome, but there is evidence highlighting different genetic and neurochemical associations with hallucinations and delusions, respectively (discussed below). Research further suggests differences between individuals with delusions who do or do not have concurrent agitation [20]. People with agitation often have more persistent symptoms and a better response to therapies (see treatment section), although it is unclear whether this is a different group phenomenologically or whether the agitation is a marker of greater severity. Literature suggests that people with delusions who do not have agitation are more likely to also have hallucinations.

There is increasing evidence that the presence of mild behavioural impairment (MBI), which includes psychosis, is a risk factor for and possibly a prodrome to AD [11•, 21]. In this context, exclusion of psychosis pre-dating dementia may be unhelpful, particularly for case/control research studies where false negatives should be minimized, although it remains important to exclude individuals with longstanding functional psychoses throughout adulthood. Accordingly, new criteria reflecting this are being proposed by the Alzheimer’s Association International Society to Advance Alzheimer’s Research and Treatment (ISTAART).

## Impact and Outcomes of Psychosis

Psychotic symptoms are frequently distressing to the individual and their caregivers and associated with poorer disease outcomes [22]. In a landmark study, Stern et al. reported that psychosis was associated with more rapid cognitive decline [23]. A review of 55 studies examining AD with psychosis found that 20 of 30 studies with cross-sectional assessments found greater cognitive impairment in AD with psychosis than without [17]. It highlighted that all 9 studies evaluating the link between psychosis and cognitive decline supported the initial observation of more progressive cognitive impairment in the presence of psychosis. More recent studies have confirmed this association and shown similar impact in MCI [14–16, 24]. Importantly, the association between AD psychosis and cognitive decline does not appear attributable to other factors including age, age of AD onset, AD duration, sex, race, education and family psychiatric history [17]. Notably, several studies have suggested that among people who develop psychosis, there is a sharper trajectory of decline even prior to the onset of frank psychotic symptoms, with one study finding that more rapid cognitive decline was present a year before the development of psychosis [16, 25, 26]. This consistent and building evidence suggests that there may be a different underlying biological and/or genetic predisposition in these individuals, and the presence of psychosis represents a more severe AD phenotype [27].

Psychosis is also associated with more rapid progression of functional impairment, hospital admissions, earlier admission to institutional care and increased mortality [14, 16, 24, 28, 29]. It has also been reported that initial psychosis is associated with increased subsequent dependency, independent of cognitive decline. In addition, psychotic symptoms are often antecedent to or comorbid with other neuropsychiatric symptoms like agitation, aggression and depression, further adding to the impact on the individual and others [20, 30].

## Genetic Correlates

The observation, replicated across studies and cohorts, that the risk for psychosis in AD runs in families, with an estimated heritability of 61% (which compares with 81% for schizophrenia and 50–60% for AD itself), provides compelling evidence that AD psychosis may be strongly influenced by genetic variation [30–36]. To date, only one small genome-wide association study (GWAS) of psychosis in AD has been reported, and no single nucleotide polymorphism (SNP) demonstrated genome-wide significance, although evidence suggested an association with an aggregated genetic risk score comprising multiple loci [37]. A follow-up study provided independent replication of a polygenic association of common variants with AD psychosis, although no individual SNP reached genome-wide significance [38]. The strongest associations were with three SNPs within the antisense transcript, RP11-541P9.3, a likely regulator of CCNG1 expression. CCNG1 may act to inhibit tau phosphorylation by cyclin-dependent kinases [39, 40]. This study found that polygenic risk for schizophrenia was protective against psychosis risk in AD, although opposing findings exist which implicate genetic liability of schizophrenia [41]. It should be noted that neither study nor the majority of candidate gene association studies have found psychosis risk to be influenced by APOE4 genotype [42].

Another small GWAS of structural copy number variants (CNV) with AD psychosis has been reported [43]. Although total CNV burden did not differ between AD cases with and without psychosis, a duplication in the APC2 gene demonstrated genome-wide significant association with AD psychosis (OR = 0.42; *P* = 7.2 × 10–10) [42]. CNVs in three other genes, SET, JAG2, and ZFPM1, suggested association with AD psychosis risk. While intriguing, all of these findings require replication in larger cohorts, and it is important to note that all have been conducted in cohorts of European ancestry, so findings are not generalizable to other populations. To date, no study has reported an association from exome or whole genome sequencing with AD psychosis, but these approaches will be required for the detection of rare, highly penetrant variants which confer risk or protection.

## Neurobiological Underpinnings

There is an urgent need for effective treatments to manage psychotic symptoms in AD, but there remain no licenced therapies. A rational approach to therapy requires better understanding of potential neurobiological mechanisms. Key postulated neural mechanisms of AD psychosis that represent potential treatment targets include the following: (1) dopamine D3 receptors, (2) serotonin (5HT), (3) cholinergic muscarinic receptors, (4) tau protein and (5) kalirin.

### Dopamine D2/3 Receptors

Post-mortem work has indicated a relationship between higher dopamine D3 receptor density in the nucleus accumbens and AD psychosis [44]. These findings are supported by a PET study using [11C] raclopride, in 21 people with AD, which highlighted significantly higher striatal D2/D3 receptor availability in AD patients with delusions compared with those without [45]. It is perhaps noteworthy that the last published major GWAS of schizophrenia (in 36,989 cases and 113,075 controls) implicated the dopamine receptor 2 (DRD2) gene [46]. Although it is important to interpret candidate gene studies cautiously, and not every study confirms an association, the balance of evidence suggests a link between D3 polymorphisms and increased risk of AD psychosis, although this will need to be re-evaluated in the context of emerging GWAS studies [42]. Some current atypical antipsychotics, such as aripiprazole, have increased D3 potency, and interestingly aripiprazole is the only historical atypical antipsychotic to demonstrate significant benefit in the treatment of AD psychosis in a single RCT [47]. Attempts have been made to develop more selective D3 antagonists (e.g. S33138, RG15, cariprazine), but none have progressed to clinical trials in AD psychosis.

### Serotonin (5HT)

A series of post-mortem reports have suggested a reduction in serotonin (5-HT) in the ventral temporal cortex and prosubiculum in AD psychosis compared with AD without these symptoms [48–50]. Other post-mortem studies indicated lower cell counts in dorsal raphe nucleus in AD patients with psychosis, potentially explaining the reduced 5HT [51]. A 2009 review highlighted that 6 of 9 studies examining 5HT2A polymorphisms reported a significant association with psychosis, but not with agitation/aggression or depression [52]. This evidence will need to be reviewed as additional GWAS studies emerge. With emerging ligands, there are opportunities to build on this work with PET studies. Of note, genetic polymorphism studies highlight a similar relationship between psychosis and 5 T polymorphisms in dementia with Lewy bodies (DLB) [53].

### Cholinergic Muscarinic Receptors

Psychosis in AD has been associated with higher muscarinic M2 receptor density in orbitofrontal gyrus and temporal association cortex than AD patients without psychosis [54]. Other post-mortem work has suggested loss of [3H]4-DAMP binding (a ligand to M1, M3, M4 and M5 receptors) in AD psychosis, contrasting with work in DLB which reported that delusions are associated with upregulation of M1 receptors [55, 56]. Post-mortem studies have consistently suggested a link between psychosis and an altered monoamine-cholinergic balance in both AD and DLB [49, 57]. Overall there is strong evidence linking changes in muscarinic receptors in AD and DLB, and further refinement is needed to understand the optimal treatment targets.

### Tau

Tau pathology is one of the most widely studied neurological correlates of AD psychosis. A number of studies have shown it to be associated with increased neurofibrillary tangle density and concentrations of phosphorylated tau in the neocortex, frontal cortex and CSF relative to AD without psychosis [58–61]. There is also work suggesting that the frequency of psychosis and persistence of psychotic symptoms are greater in AD patients with the extended MAPT tau haplotype [62]. On balance, this supports the potential utility of anti-tau agents, particularly for psychosis, of which there are a number in development for AD itself.

### Kalirin

Kalirin, a rho guanine nucleotide exchange factor, involved in dendritic spine growth has been implicated in schizophrenia. Recent studies have shown that reductions in kalirin are associated with improvements in psychosis-associated behaviours in AD mouse models, supporting earlier findings in post-mortem tissue [63, 64•]. Notwithstanding the challenges and complexities of assessing the AD psychosis phenotype in animal models, these findings indicate novel pathways, such as synaptic resilience, which warrant further investigation [65]. They also highlight the value of preclinical testing in identifying such mechanisms.

## Potential Factors Exacerbating Psychosis in AD

### Individuals with Dementia Factors

Factors that may exacerbate AD psychosis include acute medical conditions (e.g. pain, infection, dehydration), medication side effects or drug-drug interactions, unmet needs (e.g. fear, lack of sleep, boredom) and pre-existing psychiatric illness (e.g. bipolar disorder, schizophrenia) [66].

### Caregiver Factors

There is ample evidence that BPSD-like psychosis may be triggered or exacerbated when a caregiver is stressed or depressed. Other caregiver factors that may worsen psychosis include negative communication styles (e.g. anger, screaming, negative affect), critical management styles and mismatch of caregiver expectations with the stage of illness [67].

### Environmental Factors

Stress may be caused by changes in routine, too many competing or misleading stimuli, lack of stimuli, physical and environmental challenges and demands that exceed functional ability [68••].

## Current and Future Approaches for Treating Psychosis

**Fig. 1:**
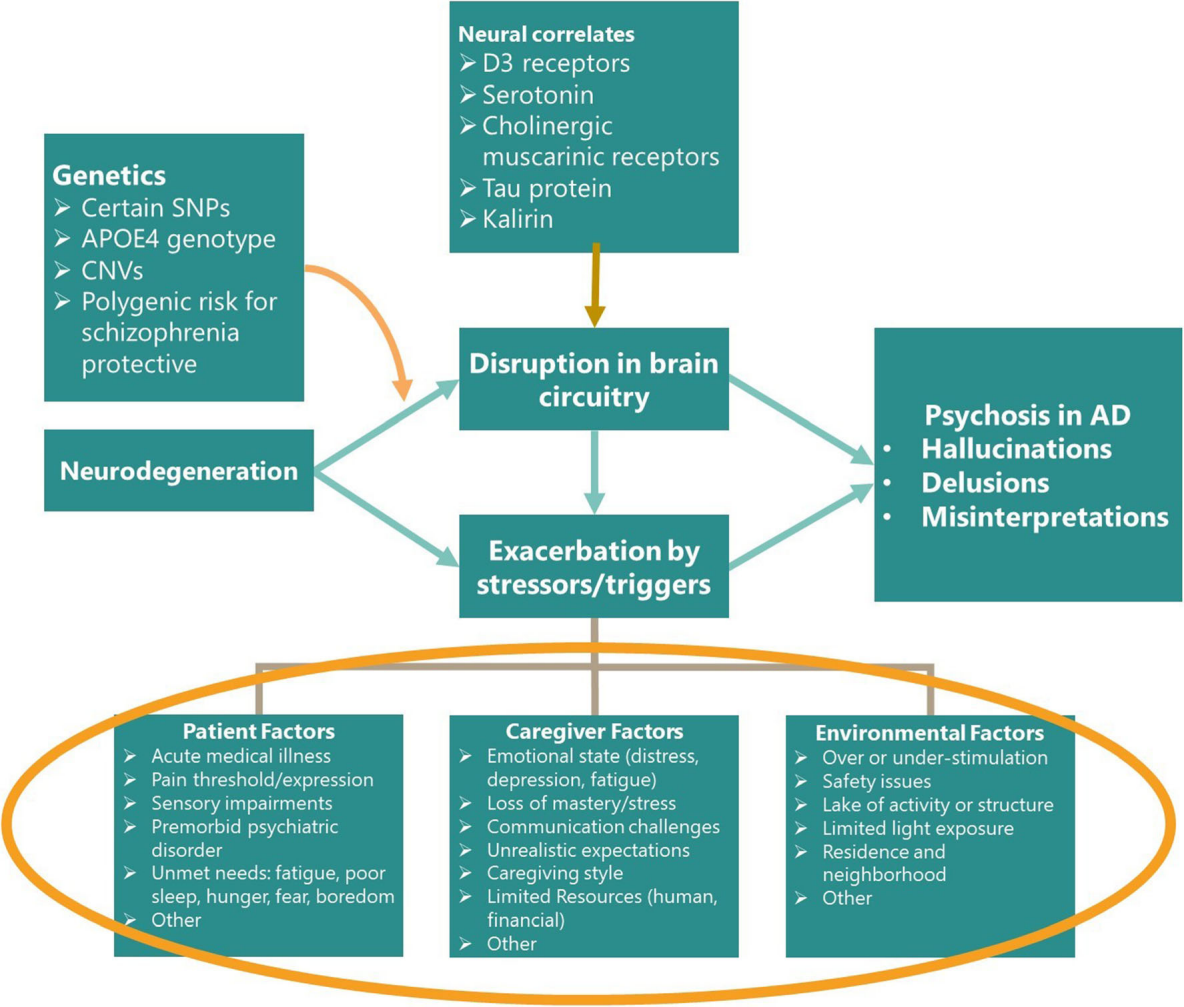
Conceptual model of psychosis in AD

Best practice guidelines, including a recent international consensus panel, emphasize the importance of investigating and treating underlying causes such as delirium, drugs increasing psychosis risk and sensory impairments [68••]. New operationalized approaches such as DICE (DESCRIBE-INVESTIGATE-CREATE-EVALUATE) [69] incorporate this into a framework enabling more consistent implementation in clinical practice. Potential underlying causes are outlined in Fig. 1.

Recently published consensus outcomes from a Delphi panel support an escalating approach to the clinical management of AD psychosis [68••]. This should start with a thorough investigation of underlying causes—which for psychosis may be delirium resulting from an untreated infection for example—and psychotropic management commenced only after consideration of other potential causes.

Second-line treatment with risperidone has been recommended. Other antipsychotics such as aripiprazole, olanzapine, quetiapine and haloperidol were not considered to have sufficient published evidence to be included in the final consensus.

### Non-pharmacological Treatments

Although there is a substantial literature to support the benefits of non-pharmacological interventions in the treatment of agitation and aggression [68••, 70], there is no RCT evidence to support the value of specific psychological or other non-pharmacological therapies for people with AD psychosis[68••]. Over the last 20 years, there have been advances in various non-pharmacological therapies for psychosis in schizophrenia and affective disorders. These include cognitive behavioural therapy and innovative approaches combining psychological therapy with technology (e.g. virtual reality) [71]. There has been minimal work to adapt this or AD psychosis, although a small series reporting CBT intervention in dementia patients experiencing psychotic symptoms suggested potential benefit [72].

Various cohort studies also demonstrate a relationship between visual hallucinations in AD and either impaired visual acuity or eye pathologies such as cataracts [72]. One study reported resolution of visual hallucinations in a small group following cataract surgery, suggesting an opportunity to improve the current treatment pathway [73].

### Pharmacological Treatments

While preclinical drug development has been limited, there has been a recent increase in RCTs of repurposed and novel agents, but less in AD psychosis than agitation. Current clinical compounds and those at various stages of clinical development for AD psychosis are reviewed below.

#### Atypical Antipsychotics

Atypical antipsychotics are the most widely used pharmacological treatment for AD psychosis. Other than pimavanserin which has a more specific mechanism of action and is reviewed in the next section, there have been no new RCTs of atypical antipsychotics in AD psychosis over the last 6 years, and the evidence has been thoroughly reviewed in previous articles [74]. A meta-analysis discussed seven trials reporting psychosis as an outcome [75]. When combined, the data from three trials with risperidone demonstrated a modest but significant improvement compared with placebo at a dosage of 1 mg per day but not at higher or lower doses (− 0.8 point in BEHAVE-AD, standardized effect size Cohen’s *d* < 0.2). Two olanzapine trials showed a nonsignificant trend towards benefit. The only other antipsychotic to demonstrate some significant benefit in an RCT is aripiprazole, in a care setting of over 400 people [47]. Risperidone is licenced in the EU, but not in the USA, for the short-term treatment of aggression in AD, but not psychosis. The modest benefits of antipsychotics in the treatment of AD psychosis need to be balanced against the substantial burden of adverse events.

The severe adverse events associated with atypical antipsychotics in AD psychosis are well established and include increased risk of parkinsonism, oedema, falls, deep vein thrombosis, pneumonia, stroke and a near 2-fold increased risk of mortality [76–79]. These side effects resulted in FDA issuing a black box warning on the use of antipsychotics in people with dementia and for similar warnings by regulatory authorities in Europe.

### Emerging Pharmacological Treatments

Recent years have seen a small but encouraging increase in the number of compounds in trials. The recent Delphi consensus highlighted pimavanserin and citalopram as the most promising potential therapies [68••].

#### Pimavanserin

Pimavanserin is a novel antipsychotic with a highly selective 5HT2A mechanism of action, lacking dopaminergic, cholinergic or histaminergic activity. Pimavanserin confers benefit in the treatment of PD psychosis and is licenced for this indication in the USA [80]. Phase II trial results of 181 patients with AD psychosis were recently published showing 55% of patients in the treatment arm experienced symptom improvement at the 6-week primary endpoint (compared with 37% in the placebo group). The adjusted mean difference for this primary outcome was − 1.84 in favour of pimavanserin, with a Cohen’s *d* standardized effect size of 0.32 comparing favourably with the 0.2 standardized effect size emerging from meta-analyses of RCTs of atypical antipsychotics [81•]. A subgroup analysis suggested that pimavanserin conferred greater benefit in people with more severe symptoms, with an adjusted mean difference of − 4.43 favouring pimavanserin (standardized effect size 0.73) in people with a combined delusions/hallucinations NPI score of ≥ 12. Caution should be exercised interpreting this exploratory analysis; however, it is an interesting finding as this more severe group is in most in need of effective treatment, and this level of benefit would clearly be clinically meaningful if confirmed in a further trial.

An exploratory analysis also suggested that participants with concurrent agitation who received pimavanserin together with a concomitant SSRI showed improved efficacy compared with those taking pimavanserin alone [81•]. This has to be interpreted cautiously as this was an exploratory analysis. However, it is consistent with previous data in PD psychosis [80], suggesting the possibility of added benefit which merits further evaluation. It is worth noting that the combination may lead to QTcF prolongation.

It is also important to note that the sustained effect of pimavanserin was unclear as there was no benefit over placebo at 12 weeks. Whether this represents an attenuation of treatment effect or a reflection of the recurring-relapsing course of symptoms remains to be established. A phase III relapse prevention trial of pimavanserin with a longer follow-up period is now underway and may address these issues.

The phase II trial also evaluated safety. QTcF prolongation (< 10 ms with no associated adverse events) was observed in the treatment group. This is consistent with the caution in the current US label for pimavanserin in PD. In contrast to other antipsychotic drugs, there were no increases in sedation, parkinsonism, stroke, accelerated cognitive decline, haematologic disorder, cardiovascular events or other significant safety risks including sudden death, . In this study, there were 4 deaths in the pimavanserin group and 4 in placebo.

A phase 3 study of pimavanserin has recently been completed using a relapse prevention design, and incorporating participants with AD, DLB/PDD, vascular dementia and fronto-temporal dementia has recently been completed. Participants received a 2 week non-pharmacological intervention, with non-responders progressing to 12 weeks of open-label treatment with pimavanserin. One hundred ninety-four (62%) of participants with a significant treatment response progressed to a double-blind withdrawal phase over 6 months, where participants were randomized to continue pimavanserin or to placebo. Over the 6 months of the double-blind phase, there was a 65% highly significant reduction in relapse among participants receiving pimavanserin, with a good tolerability profile and only 1 death in the group receiving pimavanserin. So far these results have only been published in abstract linked to a conference presentation (https://www.neurologylive.com/clinical-focus/in-dementia-pimavanserin-significantly-reduces-psychosis-relapse), and we await the full publication of the results.

Although pimavanserin remains investigational for AD psychosis, it is licenced in the USA for PD psychosis. Similar to other antipsychotic drugs, pimavanserin received a black box warning for mortality risk when it was approved by FDA. In recent media reports, its safety in PD psychosis has come under scrutiny due to a perceived increase in death rate. These include a CNN report citing 700 deaths registered in FDA’s adverse event reporting system (FAERS) as well as a report from the Institute of Safe Medical Practices (ISMP) that identified 244 deaths during the first 6 months after approval. It is difficult to draw conclusions from these databases, given that these are spontaneous reports and the inherent lack of control over how events are reported. Based on the available safety data, the FDA has recently concluded that there are no new safety concerns, but continue to monitor this key issue (https://www.fda.gov/drugs/drug-safety-and-availability/fda-analysis-finds-no-new-or-unexpected-safety-risks-associated-nuplazid-pimavanserin-medication).

Another important consideration is the potential increased mortality risk when pimavanserin is prescribed in conjunction with an atypical antipsychotic. The only published data addressing this is a long-term open-label safety study in PD psychosis [82]. In this study, 66 participants had other antipsychotics added to their regimen, and the safety profile was retrospectively compared with the 357 who remained on pimavanserin alone. There were 18.8 deaths per 100 patient-years in the group who added another antipsychotic compared with 4.5 deaths per 100 patient-years in those who remained on pimavanserin alone. In addition, there was almost a threefold increase in serious treatment-emergent adverse events in the group receiving an atypical antipsychotic. The combination of pimavanserin and an atypical antipsychotic is therefore an important issue for PD psychosis patients but may also be an issue for patients with AD psychosis if pimavanserin is licenced for this indication.

#### Citalopram

The CITAD trial randomized 186 people with AD to citalopram (ascending to 30 mg per day) or placebo for 9 weeks, demonstrating benefits in the primary outcome for agitation [83]. More recently, an analysis of secondary outcomes suggested benefits in both delusions and hallucinations in participants receiving citalopram compared with those receiving placebo, with the best response in individuals with psychosis and agitation [84]. Although this is a secondary outcome, it merits further study. A sub-study examining the genetic associations of treatment response showed a link between both HTR2A and HTR2C receptor polymorphisms and response to citalopram, suggesting a mechanistic link for the treatment of agitation, but no such evaluation was undertaken for psychosis.

### New Atypical Antipsychotics

ITI-007 (lumateperone) and SUVN-M8036 are two novel antipsychotics with 5HT2A and D2 modulation properties. Phase III trials of ITI-007 for schizophrenia, bipolar depression (NCT03817528) and agitation in AD (NCT03249376) are underway, and a future focus on AD psychosis is possible.

A key unanswered question is whether clozapine is effective for AD psychosis. There are no RCTs or substantive open trials of clozapine in AD. Two small RCTs in people with PD psychosis suggested meaningful efficacy, but in the context of well-established safety concerns such as neutropenia and mortality [85, 86].

### Anti-dementia Drugs

There is consistent evidence across clinical trials that both cholinesterase inhibitors and memantine may have a modest but significant impact on the overall level of neuropsychiatric symptoms. Although secondary exploratory analyses have suggested potential benefit in the treatment or reduced emergence of psychosis in AD with donepezil and memantine, there is no direct RCT evidence indicating a specific benefit in the treatment of clinically significant AD psychosis [87, 88]. There is evidence that cholinesterase inhibitors confer benefits in the treatment of visual hallucinations in people with LBD [89].

### Other Emerging Therapeutics

#### MP-101

MP-101 is a novel metabotropic glutamate receptor type 2 (mGluR2) and 3 (mGluR3) agonist currently being evaluated in a phase II trial (NCT03044249) for the treatment of dementia-related psychosis, with results expected in 2021 (https://clinicaltrials.gov/ct2/show/NCT03044249). MP-101 was originally developed for bipolar disorder, but this programme was discontinued in 2016.

#### Lithium

The Lit-AD study is the first RCT of lithium for AD neuropsychiatric symptoms (NCT02129348). It primarily focuses on agitation but also examines agitation with and without psychosis, the rationale being that lithium has long been used as a therapy for other psychotic disorders in the context of bipolar disease. A small series of investigator-led case reports suggested benefit on psychosis; however, this was an exploratory analysis, so it will require a more robust evaluation for AD psychosis [90].

### Muscarinic Agonists

As discussed above, post-mortem studies suggest that changes in M1 and M2 receptors are potentially associated with psychosis (particularly delusions) in AD and other dementias. A 1997 RCT of xanomeline with a primary outcome of cognition showed a highly significant beneficial effect on psychotic symptoms; however, there was an unacceptably high discontinuation rate (> 50%, largely due to GI side effects) in the active arm [91]. However, there remains interest in using muscarinic agents for cognition. Recently, a phase I pilot tolerability study evaluating xanomeline in combination with trospium chloride showed a 46% reduction in cholinergic side effects (NCT02831231). Given the previous AD efficacy data, this novel combination may represent a step towards re-evaluating the role of xanomeline in AD. There are now also novel muscarinic compounds in the development pipeline for AD and DLB.

## Conclusion

It is encouraging to see an improving understanding of potential mechanisms underlying psychosis in people with AD and a related increase in the number and breadth of intervention studies. Further genetic studies will be critical to identify novel treatment targets and to develop a framework for effective precision medicine.
